# A Prospective Comparative Study between Implantable Phakic Intraocular Contact Lens and Implantable Collamer Lens in Treatment of Myopia in Adults

**DOI:** 10.1155/2022/9212253

**Published:** 2022-03-28

**Authors:** Mahmoud Rateb, Ahmed A. M. Gad, Dalia Tohamy, Mohamed Nagy Elmohamady

**Affiliations:** ^1^Ophthalmology Department, Faculty of Medicine, Assiut University, Asyut, Egypt; ^2^Ophthalmology Department, Faculty of Medicine, Zagazig University, Zagazig, Egypt; ^3^Ophthalmology Department, Faculty of Medicine, Benha University, Benha, Egypt

## Abstract

**Purpose:**

To compare implantable collamer lenses (ICLs) and acrylic implantable phakic contact lenses (IPCLs) in the treatment of myopia in adults, as regards refractive outcome and adverse effects.

**Methods:**

Prospective, randomized comparative study with phakic intraocular lenses (IOLs) was carried out for treatment of myopia. Patients were randomized into two groups: one for ICL and the other for IPCL. Preoperative assessments included a full examination, pentacam, endothelial cell count, and biometry. We compared the adverse effects and refractive outcomes between both groups. The study was registered in clinical trials and the registration number is NCT04624035.

**Results:**

Sixty eyes of sixty patients (28 in the ICL group and 32 in the IPCL group) with a follow-up period of 12 months. The mean preoperative spherical equivalent was −12.7 ± 3.4 D and −13.6 ± 4.4 D in the ICL and IPCL groups, respectively (*P*=0.37). The mean postoperative spherical equivalent value was ±0.4 ± 0.2 D and ±0.6 ± 0.1 D in the ICL and IPCL groups, respectively. Uncorrected visual acuity (UCVA) has improved from 1.3 ± 0.06 to 0.15 ± 0.02 Log MAR in the ICL group (*P* < 0.001) and from 1.3 ± 0.02 to 0.15 ± 0.01 Log MAR in the IPCL group (*P* < 0.001). The mean endothelial cell count was reduced by 3.3% in the IPCL group and by 3.2% in the IPCL group.

**Conclusion:**

Both ICL and IPCL are effective methods to correct high myopia in adults with no statistically significant differences between the two lenses as regarding adverse effects.

## 1. Introduction

Phakic intraocular lenses (pIOLs) of different designs and materials have been used effectively instead of corneal refractive surgery in certain situations [1]. Moreover, the pIOL exhibits several advantages, as it is suitable for high myopia, with lower production of aberrations and superior contrast sensitivity [2–4]. Ordinarily, keeping accommodation is its definite advantage [5].

The Visian implantable collamer lens (ICL; Staar Surgical, Monrovia, CA), a posterior chamber pIOL, has been shown to be useful for the correction of high myopia [4–6]. Nevertheless, as an intraocular procedure, it is associated with a risk of complications such as probable injury to the anterior segment, retinal detachment, and endophthalmitis [7].

The implantable phakic contact lens (IPCL V2, Care Group Sight Solutions, India) has been developed as an alternative to ICL, with a noticeable financial advantage. Furthermore, the ICL has a power range up to −18.0 D, while the IPCL is available up to −30 D [8].

Previous studies have assessed the safety and efficiency of ICL implantation [1, 2, 4, 5, 9]. Other studies evaluated various devices for anterior segment imaging postoperatively and identified changes in the anterior segment after surgery [6, 10, 11]. A study determined the safety of IPCL over a minimum follow-up period of 1 year [8]. In this study, we aimed to compare the refractive results and adverse effects of IPCL and ICL in the treatment of myopia in adults.

## 2. Patients and Methods

This is a prospective, randomized, comparative study of myopic patients assigned for pIOL implantation. The study was approved by the Institutional Review Board of Assiut University, Egypt. Written informed consent was obtained from all patients. All procedures were performed according to the guidelines of the Declaration of Helsinki and its updates. The trial was registered in Clinical Trials. Its registration number is NCT04624035 [12].

This study was conducted at three private centers: Tiba Eye Center, Assiut, Egypt; Masa Eye Center, Benha, Egypt; and Alpha Vision Center, Zagazig, Egypt. Patients were recruited started in September 2020.

The study included 60 eyes of 60 patients who underwent pIOL implantation for correction of myopia. Inclusion criteria were age over 18 years with at least one year of stable refraction, myopia of more than six diopters, refractive astigmatism within three diopters with no other ocular or systemic disease, central anterior chamber depth (ACD) >2.8 mm (measured from the corneal endothelium to the anterior lens capsule), and an endothelial cell count ≥3000 cell/mm2. We included one eye from each patient for statistical purposes. Eyes were randomized by the sealed envelope method into two groups: the ICL group with ICL-implanted and the IPCL group with IPCL implanted. We offered the patient two sealed envelopes, and the patient chose one. The specifications of both ICL and IPCL, as found in the manufacturer's brochure, are listed in Table 1.

### 2.1. Preoperative Assessments

First, a full ophthalmic examination, pentacam, and biometry were performed. Essential measurements for pIOL implantation were performed by an experienced surgeon. Then, anterior segment ocular coherent tomography (OCT) was used to measure internal ACD and confirmed by IOL Master ACD with consideration of corneal thickness. The IOL calculation was performed according to the manufacturer's guidelines. ICL power was calculated using the modified vertex formula. Meanwhile, IPCL power was calculated using the online IPCL calculator. The size of the pIOL was selected according to WTW and ACD.

Refraction was measured objectively using an autorefractometer (Topcon KR-800, Japan), then refined to the BCVA subjectively using the trial frame. For statistical purposes, visual acuity was transformed to Log MAR. Endothelial cell counts were performed preoperatively using a specular microscope, CEM-530 (Nidek, Aichi, Japan). Intraocular pressure (IOP) was measured using an air puff tonometer (Topcon, CT-80, Japan) and was noted as normal (8–21 mmHg), low (below 8 mmHg), or high (above 21 mmHg).

### 2.2. Surgical Procedures

#### 2.2.1. ICL Group

After mydriatic eye drops and topical anesthesia instillation, a 3-mm temporal corneal incision was made. The viscoelastic material was injected into the anterior chamber (AC). An injector cartridge (STAAR Surgical) was used to insert the ICL V4c model with a central hole. The four footplates of the ICL were positioned on the ciliary sulcus along the 180° axis. The viscoelastic material was removed entirely.

#### 2.2.2. IPCL Group

Topical anesthetics and mydriatic agents were administered before surgery. The IPCL (V2 model with a central hole) was implanted into the AC through a 3 mm clear corneal incision after viscoelastic material injection. Consequently, the footplates were tucked behind the iris, followed by a thorough viscoelastic removal.

### 2.3. Postoperative Care and Follow-Up

Postoperatively, tobramycin 0.3% dexamethasone 0.1% (Tobradex) and moxifloxacin 0.5% (Vigamox) eye drops were administered topically three times daily for 2 weeks. Patients were followed up on the first day, first week, and every two months for 12 months. Specular microscopy was performed 12 months postoperatively. We compared the adverse effects and refractive outcomes between the groups.

### 2.4. Statistical Analysis

The data were examined, coded by the researchers, and analyzed with SPSS version 21 (IBM, Armonk). Descriptive statistics such as means, standard deviations, medians, ranges, and percentages were calculated. Test of significance: the chi-square test was used to compare the difference in the distribution of frequencies among different groups. For continuous variables, an independent *t*-test analysis was carried out to compare the means of normally distributed data. A paired sample *t*-test analysis was performed to compare the means of the repeated measure data. For repeated analysis of more than two intervals, repeated measures ANOVA was performed. Statistical significance was set at *P* ≤ 0.05.

## 3. Results

This study included 60 eyes of 60 patients randomized into two groups: the ICL group, 28 eyes of 28 patients with ICL implanted, and the IPCL group, 32 eyes of 32 patients with acrylic IPCL implanted. The mean age of the patients was 24.71 ± 3.3. Females accounted for 73.3% of the cases. The mean preoperative refractive error was −13.17 ± 3.9, diopters of myopia and −2.04 ± 0.9 diopters of astigmatism. The mean preoperative UCVA and the mean preoperative best corrected visual acuity (BCVA) were 1.3 ± 0.02 and 0.2 ± 0.01 LogMAR, successively (Table 2). There was no statistically significant difference between the ICL and IPCL groups in terms of age, sex, preoperative refractive error, and preoperative visual acuity (Table 2).

As for the mean refractive error in both groups, there was a significant reduction in postoperative myopia and astigmatism. There was no statistically significant difference between refraction in the ICL and IPCL groups at all postoperative visits (Table 3). The UCVA and BCVA improved significantly in the postoperative visits compared with the preoperative UCVA and BCVA. There was no statistically significant difference between UCVA and BCVA in the ICL and IPCL groups at all postoperative visits (Table 4).

The mean endothelial cell count was reduced by 3.3% and 3.2% in the IPCL and ICL groups, respectively, with no significant difference between the two groups.

As for complications, one case in the IPCL group showed lens opacity, which did not affect the final UCDVA. None of the patients had an elevated IOP.

## 4. Discussion

Posterior chamber phakic intraocular lens implantation shows several advantages over keratorefractive methods for high myopic correction. This procedure provides better optical results, and there is no risk of regression. However, this technique is invasive, with increased susceptibility to complications such as cataracts, infections, and endothelial cell loss.

The use of the ICL is restricted by its increased cost, especially in developing countries. Conversely, the IPCL is a feasible alternative for refractive correction [13]. Accordingly, we conducted this study to compare the outcomes of acrylic IPCL and ICL as options for the management of high myopia. We implanted ICLs in 28 eyes and IPCLs in 32 eyes. We followed our cases for 12 months. Both ICL and IPCL were found to be able for correcting high myopia.

Developing cataracts and elevated IOP are the most frequently recorded complications associated with phakic PCIOL implantation [7]. In our study, one case in the IPCL group showed demonstrable lens opacity, which did not influence the final UCVA. This is better than the previously reported results of Sachdev et al., who used non-holed IPCL [8]. The advantage of Hole ICL, which may decrease the risk of cataract formation, is the possible circulation of the aqueous humor through the hole, reducing the risk of lens malnutrition [14].

None of our patients had an elevated IOP during the follow-up period as holed ICLs and holed IPCLs were used. In the implantation of conventional ICLs, surgeons perform preoperative Nd: YAG iridectomies to minimize increases in the IOP [15]. This procedure is commonly accompanied by pain, especially in young patients, or with intraoperative iris hemorrhage and increased IOP. However, the central hole in the optic of the V4c Visian ICL and V2 IPCL permits near-normal aqueous humor circulation, and Nd: YAG iridotomies are not required [16].

Endothelial cell loss was 3.3% in the IPCL group and 3.2% in the ICL group, with no significant difference between the two groups. This is comparable to previously published data, with a mean ECD loss ranging from 0.3% to 7.8% in the previous studies [8, 17–19].

In this study, a significant reduction in myopia and astigmatism was observed postoperatively, with no statistically significant difference between the refraction in the ICL and IPCL groups. Significant improvement in UCVA and BCVA was detected postoperatively, with no significant difference between the two groups. This is in line with the results reported by Sachdev, GS, and associates. They retrospectively investigated eyes that underwent phakic IPCL or ICL implantation with a minimum follow-up period of 1 year [13].

This study has some limitations. The small sample size may have been inadequate to achieve statistically significant differences between the two groups for each IOL. However, the small differences between groups for outcome measures suggests this limitation did not affect the overall study results. Another limitation is that the quality of vision was not measured, which may have been able to detect any differences in optical quality between IOLs. A study with a longer follow-up (3–5 years) is required to monitor the incidence of posterior capsule opacification in both phakic IOLs.

We concluded that both ICL and IPCL are effective tools for the management of high myopia in adults. After 12 months of follow-up, there were no statistically significant differences between the two lenses in terms of efficacy and complications.

## Figures and Tables

**Table 1:** Specification of ICL and IPCL.

	ICL	IPCL
Material	Hydrophilic copolymer (collamer)	Reinforced acrylic with medium water content
Design	Plate haptic	Plate haptic
Vault	Central anterior	Central anterior
Optic hole	Central	Central and 2 others
Optic diameter	4.9–5.8 mm	6.2 mm
Widest length	12.1, 12.6, 13.2, and 13.7 mm	11–14 mm (in 0.25 mm steps)
Power range	−3.0 to −18.0 diopter	−3.0 to −30.0 diopter
Manufacturer	STAAR, Nidau, Switzerland	Care Group, Baroda, India

**Table 2:** Baseline data comparisons between the study groups.

	ICL (*n* = 28)	IPCL (*n* = 32)	*P*value
Age/years	25.14 ± 3.6	23.94 ± 2.7	0.154
Sex			
(i) Male	8 (28.6%)	8 (25%)	
(ii) Female	20 (71.4%)	24 (75%)	0.438
Eye			
(i) Od	16 (57.1%)	14 (43.8%)	0.219
(ii) OS	12 (42.9%)	18 (56.2%)	
Refraction at baseline			
(i) Sphere	−12.7 ± 3.4	−13.6 ± 4.4	0.368
(ii) Astigmatism	−2.3 ± 1.2	−1.8 ± 0.7	0.071
Visual acuity at baseline			
(i) UCVA in LogMAR	1.3 ± 0.06	1.3 ± 0.02	0.732
(ii) BCVA in LogMAR	0.2 ± 0.03	0.2 ± 0.03	0.969

**Table 3:** Preoperative, 1, 3, 6, and 12-months postoperative refraction in eyes undergoing implantable ICL vs. IPCL.

		ICL (*n* = 28)	IPCL (*n* = 32)	P1 value
Refraction				
(i) Sphere	(1) Preoperative	−12.7 ± 3.4	−13.6 ± 4.4	0.368
	(2) 1 month	0.1 ± 0.1	0.5 ± 0.1	0.221
	(3) 3 months	−0.2 ± 0.2	−0.2 ± 0.1	0.669
	(4) 6 months	−0.6 ± 0.2	−0.7 ± 0.1	0.338
	(5) 12 months	−0.4 ± 0.2	−0.6 ± 0.1	0.167
P2 value		<0.001^*∗*^	<0.001^*∗*^	

(ii) Astigmatism	(1) Preoperative	−2.3 ± 1.2	−1.8 ± 0.7	0.071
	(2) 1 month	−1.6 ± 0.1	−1.4 ± 0.1	0.168
	(3) 3 months	−1.5 ± 0.1	−1.4 ± 0.1	0.195
	(4) 6 months	−1.4 ± 0.1	−1.4 ± 0.1	0.208
	(5) 12 months	−1.4 ± 0.1	−1.3 ± 0.1	0.221
P2 value		=0.002^*∗*^	=0.013^*∗*^	

P1 between the two groups; P2 between preoperative and postoperative visits.

**Table 4:** Preoperative vs. postoperative patient's visual activity in eyes undergoing implantable ICL vs. IPCL.

		ICL (*n* = 28)	IPCL (*n* = 32)	P1 value
Visual acuity in LogMAR				
(i) UCVA	(1) Preoperative	1.3 ± 0.06	1.3 ± 0.02	0.732
	(2) Postoperative	0.76 ± 0.2	0.74 ± 0.1	0.793

P2 value		<0.001^*∗*^	<0.001^*∗*^	
(ii) BCVA	(1) Preoperative	0.2 ± 0.03	0.2 ± 0.03	0.969
	(2) Postoperative	0.15 ± 0.01	0.15 ± 0.01	0.600
P2 value		<0.001^*∗*^	<0.001^*∗*^	

P1 between the two groups; P2 between preoperative and postoperative visits.

## Data Availability

The data used to support the findings of this study are available from the corresponding author upon request.
